# Action Prediction Allows Hypothesis Testing via Internal Forward Models at 6 Months of Age

**DOI:** 10.3389/fpsyg.2018.00290

**Published:** 2018-03-12

**Authors:** Gustaf Gredebäck, Marcus Lindskog, Joshua C. Juvrud, Dorota Green, Carin Marciszko

**Affiliations:** Department of Psychology, Uppsala University, Uppsala, Sweden

**Keywords:** internal model, pupil dilation, prediction, action, interaction, eye tracking

## Abstract

We propose that action prediction provides a cornerstone in a learning process known as internal forward models. According to this suggestion infants’ predictions (looking to the mouth of someone moving a spoon upward) will moments later be validated or proven false (spoon was in fact directed toward a bowl), information that is directly perceived as the distance between the predicted and actual goal. Using an individual difference approach we demonstrate that action prediction correlates with the tendency to react with surprise when social interactions are not acted out as expected (action evaluation). This association is demonstrated across tasks and in a large sample (*n* = 118) at 6 months of age. These results provide the first indication that infants might rely on internal forward models to structure their social world. Additional analysis, consistent with prior work and assumptions from embodied cognition, demonstrates that the latency of infants’ action predictions correlate with the infant’s own manual proficiency.

## Introduction

When observing a goal-directed action, infants and adults can disengage from an ongoing event and look into the future toward locations that will soon become interesting, as goals of other people’s actions (Gredebäck and Falck-Ytter, 2015). When, for example, we observe someone eat, we do not continuously fixate on the spoon as it moves back and forth between the plate and other person’s mouth; we instead make a future-oriented saccade to the mouth prior to the arrival of the spoon (Kochukhova and Gredebäck, 2010).

The discussion as to why we predict the goal of other people’s actions has primarily focused on overcoming the temporal delay of social perception processes and the delay of the oculomotor system (Gredebäck and Falck-Ytter, 2015). It takes time to process visual information and program gaze to fixate on interesting events in the environment (usually more than 200 ms in adults; Engel et al., 1999, 250–350 ms around 1-years of age; Kenward et al., 2017, and 400–600 ms in younger infants; Gredebäck et al., 2006). Without predictive saccades we would lag behind other peoples’ actions, primarily fixating on locations where something interesting has already happened some time ago. In this context the main benefit of predictive saccades is to guide prospective attention and allow foveal vision to stay on target.

It has been argued that infants from 6 months of age predict the goal of eating (Kochukhova and Gredebäck, 2010) and reaching (Kanakogi and Itakura, 2011) actions, though there are only two studies demonstrating this and with rather small samples, ranging from 12 to 18 infants per condition. A few months later in development, from around 10–12 months, infants predict actions that involve placing an object in a container (Falck-Ytter et al., 2006), a phenomenon that has been demonstrated across a large range of studies contributed from independent labs (for example; Rosander and von Hofsten, 2011; Cannon et al., 2012; Melzer et al., 2012). Jointly these studies suggest that action prediction emerges along with the infant’s own manual proficiency (Gredebäck and Falck-Ytter, 2015). The suggested coupling between own manual proficiency and action prediction is supported by a range of correlational studies using developmental populations (Gredebäck and Kochukhova, 2010; Kanakogi and Itakura, 2011; Cannon et al., 2012; Melzer et al., 2012; Stapel et al., 2015, 2016), active interference (Cannon and Woodward, 2008; Ambrosini et al., 2012; Costantini et al., 2012), and TMS studies with adults (Cardellicchio et al., 2013; Elsner et al., 2013).

A few months after the initiation of action-based predictions, infants start to rely on statistical regularities in the environment to make predictions; both regularities in the stimuli events as they unfold (such as high vs. low probable events in the stimulus set; Henrichs et al., 2014; Adam et al., 2016) and regularities derived from the cultural context in which infants live (Green et al., 2016), suggesting that both action and statistically based predictions are present from around 9 months of age (Henrichs et al., 2014; Green et al., 2016).

Predictions based on higher order cognitive constructs such as theory of mind (Southgate et al., 2007; Senju et al., 2011) and perceived collaboration (Fawcett and Gredebäck, 2013, 2015) have been demonstrated from 18 months. Other factors such as personal traits have also been demonstrated to affect action prediction in adolescence (those with highly callous unemotional traits are less prone to predict collaborative actions; Fawcett et al., 2016). The lack of developmental data makes it difficult to theorize about the onset of trait-based influences on action prediction.

Gredebäck and Daum (2015) suggested that action prediction might be an integrative part of a larger social perception network. According to the authors, the microstructure of social perception involves several processes, some of which precede action prediction (such as agent identification and action priming that alter covert attention), others follow action prediction. The latter is referred to as action evaluation and is an umbrella term that includes pupil dilation studies (Gredebäck and Melinder, 2010, 2011), habituation paradigms (i.e., Woodward, 2009; Loucks and Sommerville, 2012), and other protocols where infants react to an event following its completion (for example, ERP studies on action outcomes, Reid and Striano, 2008; Kaduk et al., 2016). Habituation paradigms have been used extensively in the last 30 years to detect changes in attention as a function of post-habituation manipulations (Oakes, 2010) in order to assess a wide range of psychological processes including perception, cognition, and memory early in life (Rutherford and Kuhlmeier, 2013; Banaji and Gelman, 2014). Often described as a memory-based phenomenon (Oakes, 2010), these studies primarily inform us about infants’ reactions when currently perceived events do not mimic what they repeatedly saw earlier, a form of evaluation.

In the review by Gredebäck and Daum (2015) little space was devoted to the overarching benefits of such a tightly coupled system beyond the time saving aspects of predictions and priming. Efficient timing, and the ability to focus on events as they unfold, is truly important (Falck-Ytter et al., 2006). However, it is possible that action prediction also serves another role in the social world of infants that has thus far been neglected, namely that of hypothesis testing. In this paper we conceptualize this process via the internal forward model framework (Miall, 2003; Wolpert et al., 2003; Hurley et al., 2008; Ito, 2008; Tourville et al., 2008; Friston et al., 2011; Emberson et al., 2015). Proposed as a model for motor learning (Miall and Wolpert, 1996), it relies on a constant interaction between actions, predictions of future states, and sensory feedback. A mismatch between predictions and the sensory feedback can be used to alter actions (in this case predictive eye movements) and update initial assumptions about the world (Miall and Wolpert, 1996; Gentsch et al., 2016). The notion of internal models has been expanded to include perception (Emberson et al., 2015), the mirror neuron system (Miall, 2003), speech (Tourville et al., 2008), cognition (Ito, 2008), and social perception (Wolpert et al., 2003; Hurley et al., 2008; Friston et al., 2011), but to our knowledge has not been related to predictive eye movements and/or social perception in infancy (other than as a recent theoretical suggestion in Gredebäck, in press; for a related argument with respect to infants see Staple et al., 2010, and for adults see Donnarumma et al., 2017).

More specifically, we suggest that action prediction is important for the formation of social learning because it allows an observer to fixate on a future state of the world. This explicit assumption (a fixation to a particular point) will moments later either be validated (if events happen in the place suggested by the location of gaze) or proven false (if events happen elsewhere, resulting in a reactive gaze shift to this new location). The distance between the predicted goal and the actual goal of an observed action creates an error term that is fed back into the prediction system in order to improve future action predictions in similar contexts. In the context of observing someone eat, the hypothesis that food will be brought to the mouth results in a predictive eye movement to this location. Moments later the prediction will either be validated, if the food actually enters the mouth, or falsified, if the spoon makes its way to a different location (i.e., another bowl, if the spoon is used for cooking). This mechanism might help explain why infants’ predictions initially are grounded in their own motor competences and over time become more sensitive to statistical information and more complex forms of social information (as reviewed above). Action based predictions provide a foundation for the learning process. These initial predictions are restricted to a small set of actions that infants themselves can perform (Gredebäck and Falck-Ytter, 2015). They provide a foundation for learning both when correct (a small error signal) and when incorrect (generating a large error signal). These feedback loops fine-tune and update the prediction system, creating a sensitivity to statistical information and an ever-increasing range of social processes that are developing outside the action prediction system. Error terms of predictions can, in some contexts, be reduced by taking collaboration (Fawcett and Gredebäck, 2013, 2015) and/or theory of mind (Southgate et al., 2007; Senju et al., 2011) into consideration once sensitivity to this information develops. The possibility also exists that prediction based internal models help develop sensitivity to higher order cognitive constructs by highlighting areas where the model prediction errors are large. For example, when knowledge of other peoples perspective turn out to be more informative and a better predictor of others’ actions than own knowedge about the state of the world, leading to enhanced sensitivity to mental states of others and theory of mind.

Three lines of evidence provide circumstantial support for the suggestion that action prediction and action evaluation are integrative parts of internal models early in life. First, we know that action prediction and action evaluation occur in close temporal proximity. To our knowledge only two studies have directly measured both predictive eye movements and action evaluation (surprise) at the same time, following an unexpected event and within the same paradigm (see Daum et al., 2012 for cross-sectional comparisons). Gredebäck and Melinder (2010, 2011) demonstrated that infants predicted the goal of a social interaction that involved feeding actions (one actor feed another actor pieces of banana with a spoon) and that they simultaneously dilated their pupils when the feeding action does not end at the recipient’s mouth (instead the food is placed on the back of the recipient’s hand). Action prediction is only evident from 12 months in this paradigm and only in infants that have ample experience being fed (Gredebäck and Melinder, 2010), whereas surprise reactions (assessed via pupil dilations) are observed already from 4 months of age (Gredebäck and Melinder, 2011) with infants that had minimal experience being fed. These studies demonstrate that infants simultaneously make predictions and evaluate the outcome of observed events, but that these two processes are separable, with their own onset and time course.

In addition, action prediction during infancy relates to other social cognitive processes later in life. The development of predictive abilities is outlined above (Gredebäck and Falck-Ytter, 2015). Correlational studies demonstrate that action prediction in infancy is related to infants’ theory of mind at 2 years (based on a perspective taking task that does not depend on predictive eye movements; Krogh-Jespersen et al., 2015) and imitation (Gampe et al., 2016) between 1 and 3 years. This suggests that action prediction abilities early in life are related to how well children are able to reason about others’ mental states and learn from others later in life.

Last but not least, it has been demonstrated that action evaluation has an important role to play in the learning process. In their recent paper, Stahl and Feigenson (2015) demonstrated that a violation of expectations generated enhanced learning and promoted information seeking behaviors in 11-month-old infants. The associations reported by Stahl and Feigenson (2015) fits well with the notion of internal models.

Aside from the previously mentioned studies, direct evidence to support the notion of internal forward models in infancy is sparse and difficult to obtain. One way to initiate a discussion about internal models as an active component of social perception and cognition early in infancy is to formulate hypotheses based on the argumentation above. In this study we take an individual differences approach and assess relations between prediction and evaluation. If infants have internal models that guide social perception, then there should be a clear correlation between action prediction and action evaluation. According to what we refer to as the *internal model account*, both predictive eye movements and surprise reactions might be integrated through internal models and some infants may be more apt than others to learn from their environment, based on the efficiency and precision of their predictions and surprise reactions. With this proposal we do not claim that action prediction and action evaluation are one process, rather that these are two separate processes that develop together; evaluations help shape future predictions, and vice versa. An alternative *conventional account* suggests that action prediction is a time saving process with little direct connection to action evaluations (Gredebäck and Falck-Ytter, 2015). From this perspective no correlation is expected between the two processes.

The current study reports data from two separate tasks assessing action prediction and action evaluations, respectively, from the Basic Child project^1^. During the action prediction task infants observed a person eating small pieces of food with a spoon (if infants gaze arrived at the mouth before the food had arrived, then this is referred to as an instance of action prediction). During the action evaluation task infants observed an actor hand a block to another person and the block is placed either in their outreached hand (highly predictable response that should not result in pupil dilation) or on the top of their head (infants are judged to react with surprise if they respond with a larger pupil to this event)^2^. The fact that prediction and evaluation was measured in different tasks ensures that any observed association was not related to an explicit understanding of a particular context (i.e., if both responses were assessed during observation of feeding, as previously done by Gredebäck and Melinder, 2010, 2011, then a correlation might be attributed to individual differences in understanding of feeding action alone). This design allows us to assess the initial steps of a tentative internal forward model – the assumption that infants make predictions and that this ability is related to the ability to react with surprise when events do not unfold as expected, across contexts. The second step of an internal forward model – the feedback loop that alters future assumptions about the world and future predictions – is not assessed here. As this is an ongoing longitudinal study we will in time be able to assess to what degree these processes relate to imitation learning, executive functions, theory of mind, and attachment classifications, providing further assessments of the internal model account. However, this is beyond the scope of the current paper. The study also includes data from the Vineland questionnaire in order to control for general maturity.

The current study also serves a secondary purpose of assessing the robustness of action prediction at 6 months of age, the youngest age where this ability has been reported (Kochukhova and Gredebäck, 2010; Kanakogi and Itakura, 2011), as well as the notion action prediction ability is related to own motor proficiency in early infancy (Kochukhova and Gredebäck, 2010; Kanakogi and Itakura, 2011). It was noted above that the empirical support of a general ability to predict action goals at 6 months was based on a rather small set of infants (and studies). In the current study we follow a large longitudinally sample (*n* = 120 infants) from 6 to 10 months. As such, we are in a better position to assess the degree to which young infants are able to predict the goals of feeding actions and how this develops. Large-scale multi-center replication studies in the field of infancy research represent one way to achieve this goal (Frank et al., 2017; Kenward et al., 2017). Another possibility used in the current study is for individual labs to replicate prior work in high power designs. In the end, this might turn out to be a more cost effective and productive way to assess the replicability of developmental psychology research.

## Materials and Methods

### Participants

One hundred and eighteen infants visited the lab at 6 months, *M* = 185 days (*SD* = 7 days), range 170–203 days, and 110 of these same infants returned at 10 months, *M* = 302 days (*SD* = 9 days), range 289–326 days, as part of a longitudinal study (the Basic Child project). The average age of mothers at the time of the participating infants’ birth was 31 years (*SD* = 3.9; range 19–41). During the first visit all but one infant lived with both parents. The study was approved by local ethics review committee (EPN), in accordance with the 1964 Declaration of Helsinki. The study required that all parents (legal guardians) provided written consent for participation in the study. The same information was repeated verbally to the parent prior to testing. Participation required verbal consent from the visiting parent and written consent from all parents. Participating families received a gift voucher with an approximate value of 30 € as compensation for participation.

### Procedure

Children were recruited from the sample of a large epidemiological study investigating maternal health during and after pregnancy, using questionnaires at several time points during and after pregnancy. Recruitment was initiated by a question included in a questionnaire distributed at week 32 of pregnancy. The questionnaire provided information about the present study and mothers responded whether they were interested in being contacted for participation (approximately 30–50 for each month-cohort). Participants were chosen from those mothers who responded with an interest in participating. The study was conducted in a university city with generally very high educational level, therefore we deliberately over-selected mothers reporting lower education level in order to obtain a sample distribution representative of the larger population and to maximize variability. Investigating depression during pregnancy and postnatally was one of the main aims of the epidemiological study from which we recruited. We chose to over-select for mothers showing high levels of depression symptoms 6 weeks after delivery in order to study effects of depressive symptoms on child development and to increase general variability in our data. More specifically, all mothers meeting the A and C criteria for major depression according to the DSM-IV were invited to the study [in our final sample 20% met criteria A and C on the Depression Self Rating Scale (DSRS); Svanborg and Ekselius, 2003]. Exclusion criteria included serious physical health problems and prematurity (defined as 36 full weeks of pregnancy; 252 days of pregnancy). This selection was motivated by other research questions than those targeted in this paper.

The Basic Child project involves day-long sessions of approximately 4 h (including breaks and naps), focusing on eye tracking tasks, free-play with the parent, and structured interactions between infants and an experimenter.

### General Eye-Tracking Procedure

For both the action prediction and action evaluation tasks, gaze was measured with a Tobii TX300 (set to 60 Hz; Tobii Technology AB) following a 5-point calibration (Gredebäck et al., 2010). Data was analyzed using the open source analysis program TimeStudio version 3.16^3^ (Nyström et al., 2016) operated within MATLAB.

### Stimuli and Analysis

#### Action Prediction

**FIGURE 1:**
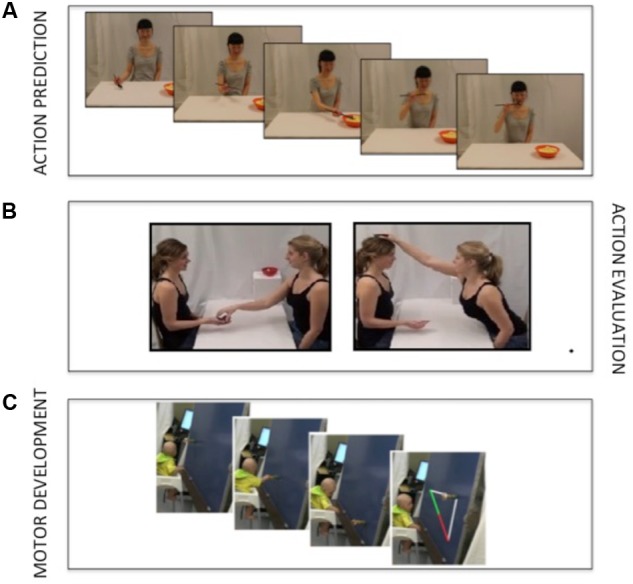
Illustrations of the action prediction task (top, **A**), the action evaluation task (middle, **B**), and motor development task (bottom, **C**).

As illustrated in **Figure 1A**, 6, and 10 month old infants observed a female model pick up pieces of food from a plate and eat them using a spoon. Each block of action prediction trials began with a 13 s movie that did not include eating actions in order to familiarize the infant to the model, the spoon, and the bowl. During the familiarization phase, the model looked up, waved, and then looked down at the spoon and said “oh,” after which she moved her spoon back and forth in order to attract attention to the tool. Between the familiarization phase and the eating action, there was a blank screen accompanied with an attention grabbing sound (“boing”) for 1.230 s. The model then reappeared and began an eating action. Each block included three repeated eating actions (all appearing sequentially following the context movie) with an average duration of 12.25 s. More information about the durations of different phases of the eating action (time from bowl to mouth, for example) can be found in Green et al. (2016). The same stimuli were used to assess action prediction at both 6 and 10 months.

Following three eating actions (each slightly different from each other) the screen once more turned black and an attention grabbing sound was played. A block of one familiarization movie and three eating actions were repeated twice in a larger eye-tracking block that also assessed perception of geometric shapes (Lindskog et al., 2015, March), gaze following (Szufnarowska et al., 2014; Gredebäck et al., 2018), and the pupillary light response to light flashes (Nyström et al., 2015).

Gaze data was exported as fixation data (Tobii fixation filter, Velocity threshold = 35 pixels/window, Distance threshold = 35 pixels). All analyses, settings, and source code can be downloaded via uwid ts-a41-88d from within the TimeStudio environment.

To assess how well infants were able to predict that the food would arrive at the mouth, we created two AIOs centered on the bowl and mouth, each measuring 6.3 × 3.8 visual degrees. The dependent variable was the time at which infants fixated within the mouth-AOI relative to when the hand and spoon first reached the mouth-AOI. Negative values indicate that infants fixated within the mouth AOI before the spoon arrived at the AOI (here defined as a predictive gaze shift). Our a priori criterion for trial inclusion was that the infant fixated within the bowl-AOI during the period from when the model first picked up a piece of food until the hand and spoon left the bowl-AOI (as defined by fixation filter). In order for a trial to be included the infant had to have fixated within the bowl-AOI and then the mouth-AOI during the period that began when the spoon left the bowl-AOI and 1 s after it had reached the mouth-AOI. Infants with less than two data points were removed from the analyses in order to achieve an approximately normally distributed dependent variable. Data were collapsed to create one prediction score per participant and age; the same was done for action evaluation and motor performance variables described below.

#### Action Evaluation

The stimuli used for the action evaluation task are illustrated in **Figure 1B**. In each video trial, 6 and 10 month old participants observed two female models seated at a table across from each other with a bowl containing wooden blocks nearest to one of the models. The trial began with a 2 s sequence in which the second actor (the ‘receiver’) performed a give-me gesture (outstretched upraised palm), while simultaneously performing “hmmm” utterance and a head-nod (to equate the amount of motion between the head and hand). After 500 ms, the second actor (the ‘giver’) then picked up one block from the bowl and passed it to either the receiver’s outstretched hand (appropriate response trials) or the top of the head (inappropriate response trials) of the receiver. Both the appropriate and inappropriate giving actions took approximately 2.5 s during which the receiver remained still with her hand extended. Once the block had been placed on the head or in the hand, the receiver placed the block onto the table, thereby finishing the trial. The entire trial lasted approximately 9 s and participants were shown 6 appropriate and 6 inappropriate trials for a total of 12 action evaluation trials. Stimuli were similar to Gredebäck and Melinder (2010) with the exception that the appropriate action was now directed toward the hand and the inappropriate action to the head (opposite of what was done by Gredebäck and Melinder, 2010). If similar effects are observed here, then the pupil dilation cannot be specifically related to a particular goal (head or hand) but rather related to the appropriateness of the action in the current context (something that was also demonstrated by Experiment 2 by Gredebäck and Melinder, 2010).

Gaze data was exported as raw data. All analyses, settings, and source code for the action evaluation task can be downloaded via uwid ts-730-9c6 from within the TimeStudio environment. Prior to extracting our measure of pupil size, the data were preprocessed in four steps. First, we removed individual eye tracking samples with a pupil size outside the range 2.5–5.5 mm. Second, samples with a change in pupil size of more than 1 mm between samples were also rejected. Third, we linearly interpolated gaps shorter than 10 samples in the eye tracking time series. Finally, we applied a moving average filter on the time series with a moving window of 10 samples.

For each trial we defined two time windows relative to when the giver grasped a block in the bowl. The baseline period began 1000 ms prior to the giver first grasping the block, after which the baseline period ended and the analysis period immediately began, lasting 3000 ms. For each trial we measured the change in pupil size from baseline to outcome. The dependent measure was calculated as the difference between the mean change in pupil size during inappropriate and appropriate trials. Our *a priori* criterion for participants’ inclusion in the final analysis was that they had data from at least three inappropriate and appropriate trials. The same stimuli were used to assess action evaluation at both 6 and 10 months.

#### Motor Development

Six month old infants interacted with a toy moving in front of them on a vertical screen (distance to the infant = 25 cm) and moving in a linear path with constant velocity (for an illustration of the trajectory of the object, see **Figure 1C**). The task has previously been used to assess how infants are able to adjust their reaching to a dynamic world (Hespos et al., 2009; for similar approaches in which the infant moves and an object is stable, see Ekberg et al., 2013) and is here considered a test of an infants’ ability to prospectively control their own actions with respect to the world – an indication of current motor development.

The task was comprised of a warm-up phase and two experimental conditions. During warm-up, a toy moved on a horizontal path back and forth in front of the infants. The toy was 3 cm wide and 13 cm long, the tip of the toy was 7 cm from the infant’s chair when directly in front of the infant. The toy moved past the infant on a trajectory that was 63 ms, moving at 30 cm/s (infant’s midline was centered with respect to the trajectory of the toy). During experimental trials the same object was placed on the left side of the infant, too high to reach. Each trial began when the infant attended to the object and had their hands close to their body. At this time the object started to move past the infant (at which point the object was within reaching distance from the infant) toward the lower right corner of the screen. Once in the lower corner (out of the infant’s reach) the object moved upward and then left to return to the starting position. If the infant removed the object from the board the object was once more placed at the starting position in order to begin the next trial. The first three experimental trials included slow moving toys (the movement of the toy past the infant extended 65 cm, infants midline halfway through the trajectory, velocity 30 cm/s) and the last three trials included fast moving toys (same trajectory, velocity 42 cm/s). The increase in difficulty over trials was implemented in order to maximize attention and interest in the task, ensuring that infants did not habituate due to initial failures at high velocities. If an infant lost interest, then the object was replaced with a new toy. Trials with parental interference or experimental error were replaced with additional trials at the end of the session.

Reaching performance was coded from three video cameras covering the entire scene (see **Figure 1C**). One camera recorded the scene from behind the infant, another camera recorded the screen and the infants from a top view, and a third camera from a side view. Researchers coded the videos frame-by-frame for: (1) whether infants attended to the object as it started to move while their hands remained close to their body, (2) the time point that the object started to move, (3) the time point in which infants started to move their hands toward the object, (4) the time point that the object reached the infants’ midline, and (5) whether the object was successfully caught or not. Individual trials were included for analysis only if infants attended to the objects and sat appropriately (consistent with criteria 1). If the hand moved at least 3 cm toward the object and stayed there or continued forward until the object had passed then this was coded as a reaching action. An additional requirement was that one of three events followed the initial reach: (1) the hand followed the path of the object as it passed the infant, (2) the object was grasped and removed, or (3) the grasp was directed to a future location of the object. The dependent variable in this task was the time point that infants initiated their reach, relative to when the object moved past the infants’ midline. Negative values indicate a predictive reach; that is, infants started to reach before the object passed the midline (consistent with prior studies; see Hespos et al., 2009). This task was only assessed at 6 months of age as reaching and grasping are well developed by 10 months (Gredebäck and von Hofsten, 2017).

### Predictions

Based on prior literature, we expected that 6- and 10-month-olds, on average, would predict the goal of eating actions and react with surprise when events unfolded in an inappropriate manner (Kochukhova and Gredebäck, 2010). Furthermore, we expected action prediction to be related to concurrent manual ability (Gredebäck and Falck-Ytter, 2015). This prediction could only be assessed at 6 months, as this is the age where manual ability was measured. Of primary importance for the current study, we additionally expected there to be a correlation between action prediction and action evaluations (Gredebäck, in press) within both ages. Action evaluations and motor ability should not correlate as prior work has demonstrated diverse developmental patterns for action prediction and action evaluation using highly similar stimuli (as reviewed above; Gredebäck and Melinder, 2011). Long-term stability is possible but not an essential component of the hypothesis being tested here, as action prediction abilities develop significantly between 6 and 10 months and are dependent on the relation between the infants’ own motor ability and the actions observed (Gredebäck and Falck-Ytter, 2015). All of these predictions have previously been presented in published papers (for more information see section “Introduction”).

### Statistical Analysis

Group level performances on the three tasks (action prediction, motor development, and action evaluation) were assessed via single-sample *t*-tests. This was followed by dependent *t*-tests in order to assess developmental differences for tasks repeated at both age groups. Preliminary analyses demonstrated that the slow trials (nr 1–3) for the motor development task was not related to action prediction or action evaluation at 6 (correlation with action prediction -0.10, action evaluation -0.10) or 10 months (correlation with action prediction 0.17, action evaluation -0.06), possibly due to the low speed of the moving object not creating sufficient task demands. These trials are subsequently removed from the analysis.

Bivariate correlation analyses (Pearson’s *r*) were conducted in order to understand associations among the included variables. For correlations, all variables were transformed so that high performance was marked by positive values. Correlations are also reported for the motor sub-scale of the Vineland questionnaire in order to account for general development (as a measure of everyday functionality, it asks what infants can do, and most importantly have been able to do, prior to the visit). It was not used as a measure of current motor development due to its low resolution (questions about fine and gross motor abilities rated by parents on a 3-point-scale) compared to the main motor task (assessing predictive reaching with an accuracy of 50 Hz). All variables were in acceptable ranges of skewness (Motor development = -0.15; Vineland = 1.59; Action evaluation 6 months = 0.47; Action evaluation 10 months = -0.78; Action prediction 6 months = -0.07; Action prediction 10 months = -0.45). Graphical inspection of all variables using Q–Q plots resulted in the assessment that all variables had acceptable distributions.

## Results

### Action Prediction

Out of the 118 6-month-old infants that participated in the longitudinal project, 42 infants at 6 months (36%) predicted the goal of the observed eating action (an average latency below zero), 37 (31%) tracked the action in a reactive manner, and 39 infants (33%) did not provide sufficient data to be included in the analysis. This means that 53% of included infants predicted the action goal. Out of the 110 10-month-olds, 74 (64%) predicted the goal, 14 (13%) were reactive, and 22 (20%) did not provide sufficient data to be included. In total, 84% of 10 month-olds included in the measure predicted the goal. Six-month-olds contributed an average of 3.5 trials (*SD* = 1.28; range 2–6 trials) whereas 10-month-olds contributed 4.3 trials (*SD* = 1.47; range 2–6 trials).

On average infants did not predict the goal of the observed eating action at 6 months [mean latency = -40 ms, *SE* = 47 ms; *t*-test against zero *t*(78) = 0.85, *p* = 0.40, negative values equal predictions]. At 10 months, however, infants predicted the same action goal [mean latency -379 ms, *SE* = 44 ms, single sample *t*-test against zero *t*(87) = 8.58, *p* < 0.0001]. Action prediction was significantly faster (more predictive) at 10 than 6 months [*t*(58) = 3,34, *p* = 0.001]. Action prediction at 6 months did not correlate with action prediction at 10 months (*r*_xy_ = 0.03; *n* = 59; *p* = 0.80).

### Action Evaluation

One hundred and fourteen infants at 6 months (97%) and 101 infants at 10 months (92%) provided data in the action evaluation task. Six-month-olds contributed an average of 5.5 (*SD* = 0.79) inappropriate and 5.6 (*SD* = 0.74) inappropriate trials, whereas 10-month-olds contributed 5.3 (*SD* = 0.96) appropriate and 5.5 (*SD* = 0.80) inappropriate trials.

On average 6-month-old infants reacted with surprise, indexed by larger pupil dilation, to inappropriate than appropriate social interactions at 6 months [mean relative amplitude = 0.063 mm, *SE* = 0.013 ms; *t*-test against zero *t*(113) = 4.91, *p* < 0.0001]. At 10 months infants no longer reacted with surprise to the inappropriate social interaction [mean amplitude -0.017, *SE* = 0.016, single sample *t*-test against zero *t*(100) = -1.07, *p* = 0.29]. Infants responses differed between the two ages with larger reactions to the inappropriate action at 6 than 10 months [*t*(97) = 4.54, *p* < 0.0001]. Action evaluation at 6 months correlated marginally with action evaluation at 10 months (*r*_xy_ = 0.19; *n* = 98; *p* = 0.06).

### Motor Performance

Forty-four 6-month-old infants (37%) demonstrated prospective control of manual reaching by initiating their reach before the moving object reached their midline. Nine infants initiated their reaches after the object had passed the midline (here termed reactive, 8%). Sixty-six infants did not provide sufficient data to be included in the analysis (55%). As such, 82% of included infants reached in a predictive manner. Data from 1.4 trials per infant (*SE* = 0.07, range 1–2) was, on average, included in the analysis. Out of these trials infants reached for the moving object on 57% (*SE* = 5.4, range 0–100). On average, infants initiated their reach before the object passed their midline, indicating predictive reaching [mean latency -380 ms; *SE* = 49 ms; single sample *t*-test against zero, *t*(52) = -7.7, *p* < 0.0001].

### Correlations

All intercorrelations are presented in **Table 1**. As expected from previous literature (Gredebäck and Falck-Ytter, 2015) action prediction at 6 months correlated significantly with motor ability at the same age. Infants that predicted the goal of others eating actions also demonstrated better prospective motor control by initiating their reach for a moving object well before it reached their midline (*r*_xy_ = 0.33; *n* = 35; *p* = 0.049), though notably this is based on approximately half the sample.

**Table 1:** Zero order correlations (Pearson’s *r*) between action prediction, action evaluation (measured at both 6 and 10 months), motor development, and the Vineland motor subscale (both measured at 6 months).

		AP	AP	AE	AE	MD	VM
		6	10	6	10	6	6
AP	6		0.03, *n* = 59	*0.34, n = 78*	0.03, *n* = 67	*0.33, n = 35*	–0.14, *n* = 79
AP	10			0.01, *n* = 84	0.14, *n* = 84	0.04, *n* = 38	–0.11, *n* = 88
AE	6				*0.19, n = 98*	0.12, *n* = 52	–0.08, *n* = 114
AE	10					0.19, *n* = 45	0.13, *n* = 101
MD	6						0.07, *n* = 53
VM	6						

*AP, action prediction; AE, action evaluation (reverse coded); MD, motor development; VM, vineland motor sub-scale. Significant correlations (p < 0.05) marked with red italic, marginally significant values (p = 0.06) marked with blue italic. This table (and the correlations between the slow motor trials, action prediction, and action evaluation noted in the methods) includes all correlations that were performed in preparation for this study.*

**FIGURE 2:**
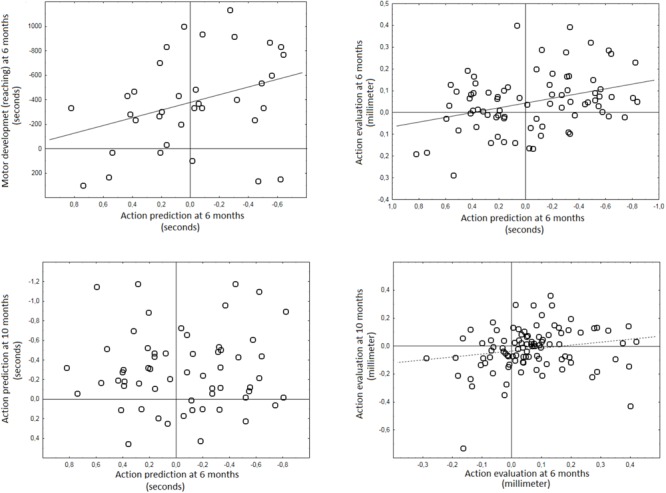
Correlations between key variables used to assess the presence of internal models in early infancy. All variables (Action prediction = latency in seconds, negative values = prediction; action evaluation = relative pupil dilation in mm positive values = surprise to inappropriate social interactions; motor performance = latency in seconds, negative values = prediction) maintain original format (variables are not flipped) as reported in prior work. Solid diagonal lines represent significant correlations (*p* < 0.05) whereas dashed line represent marginally significant interaction (*p* = 0.06). Horizontal and vertical lines mark zero-point (division between prediction and reaction for action prediction and motor development and between larger pupil to inappropriate or appropriate events for action evaluation).

More importantly, action prediction at 6 months correlated positively with action evaluation (reversed scores for this test) at 6 months (*r*_xy_ = 0.34; *n* = 78; *p* = 0.002) in accordance with the main internal model hypothesis (see **Figure 2**). No significant correlation was observed between action prediction and action evaluation at 10 months. No correlations were significant for the Vineland motor scale (see **Table 1**).

### Additional Analysis

As noted above, about half of the infants did not contribute data to the analysis of motor performance. In order to ensure that there were no differences between included and excluded infants that might have impacted the reported correlations, we conducted an additional analysis. These demonstrated that the two groups of 6-month-olds (those included and excluded on the motor performance task) did not differ in terms of general motor maturity (Vineland) at 6 months, *t*(51) = -0.70, *p* = 0.48, or in their ability to predict action goals, *t*(24) = -0.52, *p* = 0.60. Their reactions to appropriate and inappropriate social interactions also did not differ, *t*(47) = -0.03, *p* = 0.97.

## Discussion

In this study we asked the following questions: (I) can we find initial evidence to support the notion that internal models might support social information processing in infancy? and (II) can we replicate prior findings demonstrating action prediction at 6 months of age and the previously demonstrated correlation between action prediction and motor development at the same age, using a high power, longitudinal study with a large sample?

With respect to the first question, there was a clear association between action prediction and action evaluation at 6 months. This novel finding provides support for the relation between infants’ ability to predict the goal of other people’s actions and their tendency to become surprised when social interactions take unexpected turns. This correlation is present even when action prediction and action evaluation are assessed in different social contexts and using different dependent variables.

With respect to the second question, the basic principles of early action prediction are partially replicated. Many infants are able to predict action goals at 6 months of age and there is a clear connection between infants’ action prediction and their corresponding motor ability at the same age. We argue that infants’ manual ability is related to their eating skills, their ability to reach, and ability to detect the goals of others that are eating. It might be reasoned that a broad application of motor plans for reaching is unlikely; however, the notion of broad motor schemas that incorporate a wide range of similar actions early in development is well supported by the literature. Over time motor plans narrow, but this specialization occurs well above the ages tested in the current study (D’Souza et al., 2016). These findings mirror the large amount of established literature on action prediction early in infancy that connect prediction and motor proficiency, particularly with respect to observation of manual actions and infants’ own manual motor ability (Gredebäck and Falck-Ytter, 2015). The one finding that does not seem to correspond entirely to the few studies that have previously demonstrated action prediction at 6 months (Kochukhova and Gredebäck, 2010; Kanakogi and Itakura, 2011) is that only about half of the 6-month-olds predicted the goal of eating actions in the current context. Given the large sample used here we assume that this is a more accurate representation of the actual distribution than what has previously been reported. Infants at this age are just beginning to predict action goals and this ability develops until 10 months of age, when the vast majority of infants have fully developed this ability. It would have been a more desirable outcome had a larger portion of the 6 month-olds reached for the moving toy. As noted above, supplementary analyses suggest that there were no systematic differences in the key variables being assessed between reaching and non-reaching infants. Our interpretation is that some infants are simply more cautious than others, but that this does not reflect their motor ability, but rather situational or dispositional factors outside the aim of this study.

There are a few caveats to the current study that need to be acknowledged. First, it is clear from our introduction that we expected similar effects at 6 and 10 months of age; however, correlations between action prediction and evaluation were only present at the youngest age. Does this mean that internal models only impact infants at the onset of action prediction abilities at 6 months? Most likely this is not the case. The lack of correlations should instead be attributed to the fact that the majority of infants at 10 months predicted the goal of the eating action (a possible ceiling effect, as 84% of infants that contributed data predicted the goal at this age), leaving little room for correlations to be expressed. Had other contexts been examined that were more age-appropriate (such as placing balls into buckets, stimuli previously used by Falck-Ytter et al., 2006), we expect that the correlations to action evaluation would have remained.

In a similar manner, it is clear that the action evaluation task did not result in systematic pupil dilations at 10 months. Infants at this age simply do not appear to react to these events anymore. There might be several reasons for this. First, infants have previously seen the same stimuli at 6 months and therefore they are perhaps no longer equally as surprising. In addition, the current stimuli are rather short and de-contextualized (each trial is interspersed between other stimuli in order to create an interesting stimulus set to counter habituation) compared to prior studies that include longer social interactions and several repetitive actions (Gredebäck and Melinder, 2010, 2011). A de-contextualized reaching might not result in surprise in older infants as they might assume that there are unknown contextual factors that explain the action (for evidence that perceived context is important for the degree to which infants dilate their pupil in surprise, see Gredebäck and Melinder, 2010, 2011). These explanations are speculative and additional work is needed to assess the parameter space surrounding infants’ action evaluation tendencies and surprise reactions to inappropriate social interactions.

The third caveat concerns habituation and the assumed separation between processes that occur after an event has occurred and those that occur prior to the completion of an event. It is conceivable that habituation, contrary to what was suggested by Gredebäck and Daum (2015), captures infants’ expectations about what is to come and their reactions when these expectations are violated. The processes that underlie habituation and dis-habituation are not known, but have been related to memory for prior events (summarized by Oakes, 2010). These processes might include a implicit future-oriented process, but we make the assumption here that it is the act of looking at a location ahead of time that provides a concrete and non-representational form of hypothesis testing, as long as infants maintain their gaze on that location once events play out.

We argue for the presence of internal models, but it is reasonable to note that there are other complementary forms of learning that can involve both action prediction and action evaluation, such as associative learning (Niv and Schoenbaum, 2008) or sequence learning (Janacsek and Nemeth, 2012). In the current paper we do not make the claim that these other forms of learning mechanisms do not involve various degrees of prediction. We propose internal models as the mechanism behind the current results because of the close connection between motor system development and action prediction reported here and elsewhere (Gredebäck and Falck-Ytter, 2015). There is strong support for the involvement of internal models in motor planning and we suggest that similar models can also be used as part of a larger social cognitive network (in accordance with Miall, 2003; Wolpert et al., 2003; Hurley et al., 2008; Ito, 2008; Tourville et al., 2008; Friston et al., 2011; Emberson et al., 2015).

Ultimately, an update to the linear model proposed by Gredebäck and Daum (2015) is most likely needed, one that includes feedback loops and the possibility to update expectations based on the outcome of observed events and the infant’s reactions to these same events. More concretely, the inclusion of forward models require an update function where predictions are related to actual outcomes and an error signal provides a top-down modulation of prior processes.

## Author Contributions

GG, ML, and CM designed the longitudinal study (Basic Child) from which the data is taken. JJ and DG designed the action evaluation and action prediction task, respectively. GG analyzed the data and wrote the first draft of the paper. All authors provided in depth constructive comments on the paper and approved the final version.

## Conflict of Interest Statement

The authors declare that the research was conducted in the absence of any commercial or financial relationships that could be construed as a potential conflict of interest.

## Footnotes

1 This project assesses the foundation of cognition early in life using a longitudinal individual difference approach. Infants take part in procedures assessing approximately 20 constructs at 6, 10, 18, and 30 months along with a strange situation attachment scenario at 12 months.

2 Note that the current study reverse the relation between predictable and unpredictable events relative to what was previously used by Gredebäck and Melinder (2010, 2011) where the predictable event was directed to the mouth and the unpredictable event was directed to the hand. Together the two studies ensure that the any group mean differences in pupil dilations is not caused by increased attention when observing hands or faces.

3 http://timestudioproject.com
